# Auditory processing, acoustic reflex and phonological expression

**DOI:** 10.1590/S1808-86942010000600014

**Published:** 2015-10-19

**Authors:** Tiago Mendonça Attoni, Victor Gandra Quintas, Helena Bolli Mota

**Affiliations:** 1 Master's degree in human communication disorders. Speech disorders; 2 Master's degree in human communication disorders. Speech disorders; 3 Doctoral degree in linguistics and language studies, Rio Grande do Sul Pontifical Catholic University. Associate professor at the Santa Maria Federal University. Santa Maria Federal University (Universidade Federal de Santa Maria)

**Keywords:** hearing, acoustic, child, reflex

## Abstract

**Abstract:**

It is thought that a close relationship exists between auditory processing, the acoustic reflex and speech.

**Aim:**

A retrospective study to evaluate these three aspects in children with and without phonological disorders and seek any relationship among them.

**Material Methods:**

46 children were enrolled: 24 had normal speech abilities and 22 had phonological disorders. All children underwent auditory processing and acoustic reflex threshold testing.

**Design:**

Cross-sectional contemporary.

**Results:**

Auditory processing and acoustic reflex thresholds were abnormal in all children with phonological disorders. This was not the case in children with normal speech development.

**Conclusion:**

Changes in the auditory processing and acoustic reflex thresholds are closely related to speech difficulties.

## INTRODUCTION

Acquiring speech is an apparently simple process. Children quickly learn their mother tongue at a rate that does not depend on culture. They are able to discriminate and understand speech structures before speaking; this is possible because the neurophysiologic and cognitive underpinnings of speech develop before the child becomes able to issue words ^1^.

The oral myofunctional system, the central nervous system, and the auditory system should be intact for speech acquisition to occur normally. If any of these structures is compromised, children will encounter difficulties in phonological perception, organization and production.^2^, ^3^

However, there are children that present altered phonological systems with no detectable etiological factors, detected by an incorrect use of speech. Such children are labeled patients with phonological disorders.^4^, ^5^ The level of severity of phonological disorders in described by the percentage of correct consonants (PCC) given by the child; it may be classified as severe (PCC<50%), moderate-severe (50%<65%), moderatemedium (65%<85%) and medium (85%<100).^6^

In seeking answers about the factors involved in speech disorders, researchers have related phonological disorders with auditory processing conditions.^7^, ^8^, ^9^, ^10^

Auditory processing is the reading of auditory signals; its attributions are the ability to locate sources of sound, and to focus, discriminate, recognize and understand auditory stimuli. Auditory structures in the central and peripheral nervous system should operate normally for natural and ample function; otherwise auditory processing abilities will be compromised, as evidenced by disorders in receiving, analyzing and organizing auditory information.^11^, ^12^

On the function of peripheral structures, there appears to be a close relation between acoustic reflexes, stapedial muscle contraction, and auditory processing; altered acoustic reflexes indicate disordered auditory processing behaviors.^13^, ^14^, ^15^

An important tool is acoustic reflex threshold testing, which assesses efferent pathways and provides information about the brainstem.^16^ Functions include: improved auditory attention for continuous sounds; separating auditory signals from background noise; perceiving altered intensity over the auditory threshold; attenuation of noise from chewing and jaw movements during speech; vocalizing; improved speech discrimination at high intensities and selected frequencies; improved localization of sound and a sense of direction of sound by binaural interaction.^17^, ^18^, ^19^, ^20^, ^21^

The normal range of the intensity level or response threshold of acoustic reflexes is 70-90 dBHL; values over 90 or absent responses are pathological values.^22^, ^23^

The purpose of this study was to verify the auditory processing abilities and the acoustic reflex thresholds in children with normal and disordered speech development, and to correlate the severity of phonological disorder with auditory processing and the acoustic reflex; additionally, to correlate the results of acoustic reflex studies with the phonological system of children with disordered phonology.

There is a need to understand the reasons whereby some children develop speech out of the standards of their local dialect without apparent etiological circumstances. This may help clarify the causes of such disorders, the underlying physiological mechanisms of sound discrimination and acquisition, and to open therapeutic possibilities.

## MATERIAL AND METHOD

The institutional review board approved this study, which was registered as no. 23081.006440/2009-60.

The sample comprised 46 children of both sexes, aged from 5 to 7 years. There were two groups: a control group consisting of 24 children of normal speech development (8 male and 16 female), and a study group comprising 22 phonologically disordered children (12 male and 10 female).

Inclusion criteria for the control group were: no phonological disorders; no apparent neurological, emotional or perception disorders; no phonoarticulatory anatomical or physiological disorders; no language difficulties (expression and understanding); normal hearing; being right-handed; no complaints referring to auditory processing.

The exclusion criteria for this group were: anatomical or physiological disorders relating to communication; the presence of language understanding or expression disorders.

The inclusion criteria for the study group were: a diagnosis of phonological disorder; no apparent neurological, emotional or perception disorders, no phonoarticulatory anatomical or physiological disorders; no language difficulties (expression and understanding); normal hearing; being right-handed; not having undergone phonoaudiological therapy.

The exclusion criteria for the study group were any perceptible factor that could directly cause, worsen or maintain phonological disorders. Thus, only children with progressive phonological disorders were selected.

The control group was selected from a school of a philanthropic institution in the city of Santa Maria, RS. The school invited parents or caretakers to a meeting; they were given explanations about the study procedures and signed a free informed consent form.

The study group was selected from a waiting list of phonologically disordered patients.

A Fonix FA - 12 clinical audiometer with TDH 39 earphones (ANSI S3.6/96: ANSI S343/92 calibrated) was used. Stimuli were given in an acoustic booth. An AZ& impedance meter with TDH 39 earphones and 220 Hz at 70 dB probes was used for investigating contralateral acoustic reflexes at 500 to 4,000 Hz (ANSI S3.6/96: ANSI S343/92 calibration). The following tests for assessing auditory processing were applied: simplified auditory processing evaluation (with jingle bells, agogo, bells and coconut shells), the dichotic digit test, the speech-in-noise test, the staggered spondaic word (SSW) test, the pediatric speech intelligibility (PSI) test, and the posters with illustrations of responses. Volume 1 and 2 CDs were used - with test recordings - according to the operations manual.^24^

The researcher assessed both groups at a school clinic of a higher education institution.

All children underwent phonoaudiological screening, as follows: examination of the stomatognathic system, language assessment, ontological examination, audiologic evaluation (pure tone audiometry, speech reception threshold, and speech perception and recognition rate), investigation of the acoustic reflex and tympanometric curve, and a speech assessment.

The child phonological assessment tool was used to study the phonological system;^25^ with this tool it is possible to gather and analyze a significant speech sample containing all phonemic contrasts in Brazilian Portuguese and in different syllabic positions and structures.

Acoustic reflexes of the right and left ears at 500, 1,000, 2,000 and 4,000 Hz were assessed in all children. It was done ipsilaterally and contralaterally to the ear being evaluated. The normal range for our purposes was 70 to 90 dBHL; absent results or values over 90 dBHL were considered as abnormal.^23^

Auditory processing was done with tests that assessed diotic, monotic and dichotic hearing abilities.

### Diotic listening

An agogo, coconut shells, jingle bells and bells were used in the simplified auditory processing evaluation. This test requires no sophisticated mechanism, and is easily applied. It consists of three steps: localization of sound - the child should locate the source of sound, and should provide a correct answer in at least four directions out of five; errors, when they occur, should preferably be in front or above the head. The next step is verbal sequential memory: syllables are presented and the child is asked to repeat at least two of three sequences that are presented. The third step is nonverbal memory sequence: here, the child should listen to predetermined musical instrument sounds (jingle bells, agogo, bell, coconut shell) and repeat correctly the sequence; the child should be correct in two of the sequences.

### Monotic listening

The PSI test evaluated the figure-background ability and audiovisual association. Sentences with competing ipsi- and/or contralateral messages are presented; the subject is instructed to hear a story and at the same time point to one of the drawings in front of him or her.

For the speech-in-noise test, children are told that they would hear several words and noise, and that they should repeat the words. This test assesses the ability of auditory closure.

### Dichotic listening

The dichotic digit test (free and directed attention) consists of four presentations of a list of disyllable digits in Brazilian Portuguese; four different digits are presented simultaneously, two in each ear, which characterizes a dichotic task. This test evaluates selective attention abilities (directing one's hearing).

The SSW test evaluates auditory analysis/synthesis abilities and memory; it consists of identifying four different disyllables presented simultaneously to both ears. Children are instructed to repeat the four sentences presented.

The analysis included correlating the findings of auditory processing, acoustic reflexes, and speech for each group. The statistical analysis was made with the SAS (user's guide) Statistical Analysis System Institute, Inc., Cary, NC, 2001; Pearsons's coefficient correlation test.

## RESULTS

The results of the PSI and speech-in-noise tests will not be presented here, as there were no variations within and among groups; all subjects attained the maximum score in both tests. Thus, there is no reason for a statistical analysis.

Only the study group had altered results in the simplified auditory processing evaluation.

The qualitative analysis of the SSW test showed that children in the study group generally had high values pertaining to exchanges and omissions; these subjects also required more time in the analysis of behavioral responses related to temporal performance. The quantitative analysis of the SSW test showed that the control group attained normal values.

The dichotic digit test showed the number of errors in each group. In the free attention step (right and left), the response rates were similar between both ears in each group. Only study group participants had values out of the normal range.

Table 1 shows these results.

**Table 1:** Distribution of values of the simplified auditory processing evaluation, the SSW test, and the dichotic digit test.

Variables	Control group	Mean	SD	Minimum	Maximum

SI	24	2.91	0.28	2.0	3.0
SV	24	5.29	0.46	5.0	6.0
TLS	24	4.91	0.40	4.0	6.0

Variables	Study group	Mean	SD	Minimum	Maximum

SI	22	1.81	1.00	0	3.0
SV	22	4.90	1.19	3.0	6.0
TLS	22	4.04	0.95	2.0	5.0

Variables	Control group	SSW Mean	D P	Minimum	Maximum

DNC	24	6.37	5.25	0	17.0
DC	24	7.68	5.13	0	17.0
EC	24	8.68	6.46	0	15.0
ENC	24	9.25	5.29	0	17.0

Variables	Study group	Mean	SD	Minimum	Maximum

DNC	22	45.30	5.30	37.0	75.0
DC	22	65.50	3.39	40.0	75.0
EC	22	58.50	5.01	40.0	75.0
ENC	22	48.89	6.12	40.0	65.0

Variables	Control group	DD Mean	D P	Minimum	Maximum

AL	24	4.33	2.47	2.0	8.0
AOD	24	1.66	3.44	0	9.0
AOE	24	0.16	0.81	0	4.0

Variables	Study group	Mean	SD	Minimum	Maximum

AL	22	39.22	4.70	20.0	56.0
AOD	22	20.27	5.12	2.0	34.0
AOE	22	19.77	4.10	0	36.0

SI= instrument sounds; SV= verbal sounds; TLS= localization of sound test; DNC= right non-competing; DC= right competing; EC= left competing; ENC= left non-competing; SD= standard deviation; AL= free attention; AOD= attention right ear; AOE= attention left ear; DD= dichotic digit; ASPA= simplified auditory processing evaluation.

Table 2 shows the representation of ipsi- and contralateral acoustic reflex values in the study and control groups; the mean, variation coefficient, standard deviation, and minimum and maximum are presented.

**Table 2:** Results of the evaluation of ipsilateral and contralateral acoustic reflexes in the control and study groups

Variables	Control group	Mean	SD	CV%	Minimum	Maximum

			IPSILATERAL			

od500	24	84.20	7.39	8.79	75.0	90.0
oe500	24	83.10	6.40	6.71	75.0	90.0
od1000	24	82.80	6.57	7.52	80.0	90.0
oe1000	24	82.15	6.27	7.32	70.0	85.0
od2000	24	82.10	8.25	8.49	70.0	85.0
oe2000	24	83.40	7.30	8.77	80.0	90.0
od4000	24	84.20	9.05	6.93	80.0	90.0
oe4000	24	82.60	8.89	7.95	70.0	85.0

	study group					

od500	22	93.82	6.48	6.88	75.0	120
oe500	22	94.68	7.38	7.82	75.0	110
od1000	22	94.31	7.76	7.26	80.0	120
oe1000	22	95.10	7.73	6.24	75.0	110
od2000	22	94.47	7.51	7.95	75.0	120
oe2000	22	95.38	6.32	8.79	80.0	110
od4000	22	95.17	8.05	8.64	80.0	120
oe4000	22	93.24	7.89	8.76	70.0	120

			CONTRALATERAL			

	control group					

od500	24	85.70	8.23	8.89	80.0	90.0
oe500	24	84.30	7.54	7.81	80.0	90.0
od1000	24	83.20	6.57	6.25	80.0	90.0
oe1000	24	82.40	6.72	7.23	75.0	85.0
od2000	24	81.20	8.42	7.94	70.0	85.0
oe2000	24	85.60	7.23	6.78	75.0	90.0
od4000	24	82.30	8.98	8.63	75.0	90.0
oe4000	24	81.40	8.35	8.75	70.0	85.0

	study group					

od500	22	95.40	6.99	7.60	90.0	115
oe500	22	95.60	6.80	6.70	90.0	120
od1000	22	94.23	5.83	7.25	85.0	120
oe1000	22	94.22	6.84	6.40	85.0	120
od2000	22	95.51	7.74	7.86	85.0	120
oe2000	22	95.10	7.07	7.49	90.0	110
od4000	22	93.25	7.95	7.51	90.0	120
oe4000	22	93.39	6.31	6.85	85.0	115

SD=standard deviation; CV=coefficient of variation; OD= right ear; OE=left ear

Representative correlations were found in acoustic reflexes and the severity of phonological disorders. Equally significant values were found in the correlation between altered speech phonemes and acoustic reflexes. Table 3 shows these results.

**Table 3:** Results of correlations in acoustic reflex findings and the severity of phonological disorders, and with altered speech phonemes

Phonemes	Study group	AR - A	AR - P
GDF	22	0.87**	0.91***
/ p /	22	-0.21	-0.15
/ t /	22	-0.13	-0.32
/ k /	22	-0.01	-0.23
/ b /	22	0.93*	-0.17
/ d /	22	0.89*	-0.11
/ g /	22	0.95*	-0.32
/ v /	22	0.36	-0.21
/ f /	22	0.45	-0.40
/ z /	22	0.51	-0.31
/ s /	22	0.53	-0.29
/ S /	22	0.65*	0.89*
/ Z /	22	0.93*	0.92*
/ l /	22	0.13	-0.19
/ L /	22	0.95*	0.34
/ r /	22	0.49	0.39
/ R /	22	0.11	-0.12

GDF= severity of phonological disorder; AR - A=absent acoustic reflex; AR - P= present acoustic reflexes.

* Significant at P<.0.05

** Significant at P<.0.05 for moderate-severe and severe phonological disorder.

*** Significant at P<.0.05 for moderate phonological disorder.

## DISCUSSION

The lack of detectable etiologies for auditory diseases has raised attention to studies about the causes of phonological disorders. The fact that children with this condition typically have similar anatomical and physiological structures as children with normal development of speech suggests that the causative factor is present in structures that discriminate auditory stimuli.

As the first vocal sounds are produced, there is adequate feedback if auditory structures are intact, which leads to speech development.^26^ Thus, speech sounds are correctly perceived, discriminated and processed in the entire auditory complex for phonemic acquisition to take place.

Two significant events on the auditory system in children with phonological disorders were noted in this study. All children presented altered results in specific auditory processing tests. Secondly, the results of acoustic reflex testing were abnormal in all children - all values were either absent or over 90 dBHL. We noted that normal-speaking children did not have abnormal auditory processing values; their acoustic reflexes were also within normal limits. The data suggest that auditory processing and acoustic reflexes have an important role in guiding the process of acquiring speech sounds.

These findings also confirm a relation between abnormal auditory process and acoustic reflexes.^13^, ^14^, ^15^ We were unable to measure up to which point the abnormalities were associated; they are, however, closely linked to impaired speech development.

Normal and abnormal auditory processing patterns were found in study group children.

The results of the simplified auditory processing evaluation were abnormal, and there was a significant relation between non-verbal and verbal sounds. No correlation was found between performance and gender or age.

All children in both groups scored 100% in the PSI and speech-in-noise tests; this differed from a study^10^ that reported significant age-related differences in the PSI test in phonologically disordered children. Another study^27^ that applied the PSI and speech-in-noise tests children with impaired learning found significant age-related correlations.

The results of these two tests showed that the figure-background, audiovisual association and auditory closure auditory abilities were not affected. Thus, phonologically disordered children had no difficulties in integrating auditory information (integrative auditory agnosia).

The SSW test presented abnormal results; there was no best performance in any special condition, and no age or gender relationship.

Errors in the dichotic digit test did not generate significant differences between ears, notwithstanding the abnormal results in all steps.

The three tests (diotic and dichotic) showed that children had difficulties in selective attention, auditory analysis-synthesis and memory abilities. Therefore, the types of auditory processing disorders may be caused by losses in decoding and organizing auditory information.^24^. The data underline the connections between phonoarticulatory disorders and altered auditory processing, which results in incorrect use of phonemes.^28^

The correlation coefficient values on the acoustic reflex and speech in phonologically disordered children were representative. Children with preserved acoustic reflexes at all tested frequencies - over the normal threshold - had less affected phonological systems. Otherwise, absent acoustic reflex responses at some of the tested frequencies were associated with worse phonological expression.

Absent acoustic reflexes in at least one frequency appears to indicate that children find it harder to acquire sounds. The expressive phonological ability is less impaired when there are abnormal values without absent responses.

Absence of acoustic reflexes at any frequency was significant when the degree of severity of the phonological disorder was moderate-severe or severe. The severity levels of moderate phonological disorders were significantly related with the presence of increased acoustic reflex values. These results support the hypothesis that acoustic reflexes participate in the processing of sounds.^29^

The phonological component may be perceived early in child development. Acquisition of phonemes occurs gradually and periodically. Children first tend to acquire the plosive and nasal phonemes. First, the phonemes /p/, /t/, /k/. Next, the phonemes /b/, /d/ are perceived. Later, the phoneme /g/. The nasal phonemes /n/ and /m/ arise at the same time as plosive phonemes - from age 1:6 to 1:8. The nasal /ñ/ arises at age 1:7. Children acquire the fricative phonemes from age 1:8 to 2:10. The acquisition order is: /v/, /f/, /z/, /s/, /S/, /Z/. The phonemes /l/, /R/, /L/, /r/ arise from age 2:8 to 4:2, in this order.^30^, ^31^, ^32^

The phonological system was compromised if acoustic reflexes were absent. This was clear in the significance between absent acoustic reflexes and its effect on some phonemes, especially those of earlier acquisition (plosive phonemes).

An analysis of the most impaired phonemic structures and acoustic reflexes showed that the most affected sounds were voiced, requiring vocal fold vibrations.

A few authors^33^ argue that differentiating voiced from voiceless sounds is an innate ability. Babies can already discriminate some sounds at age one month. These authors state that there is a basic point at which discrimination occurs, the time at which voicing begins or voice onset time (VOT). This is the time interval between release of stop phonemes and the onset of voicing. Auditory discrimination is an important requirement for differentiating sounds; it enables children to differentiate voiced from voiceless sounds. Devoicing (children replace a voiced sound with a voiceless sound) is closely linked to a lower phonemic discrimination potential.^34^

A study^35^ that assessed VOT by acoustic analysis of phonologically disordered children showed that this ability is out of phase in these children, which may make it impossible to acquire phonemes.

Acoustic reflexes are active before and during voice. Contraction of acoustic reflexes attenuates air and bone conducted sounds, as the sound level issued by the speaker's voice that reaches the cochlea is very loud at lower frequencies, which impairs simultaneous speech reception from another person. This effect decreased the amplitude of the speech signal during voice. Attenuation of low frequency components of the speaker's sounds because of acoustic reflexes appears to affect the perception of sound and speech intelligibility.^36^

Altered acoustic reflexes may represent a hindrance at the phoneme discrimination point; one of its representations may be non-perception of the VOT. Acquisition of sounds is impaired if hearing does not return to adequate levels.

The significant association between the phonemes /S/, /Z/ and /L/ and altered acoustic reflex should be noted. The first two are fricative sounds and the latter is a lateral liquid; all are more complex sounds acquired later, and all share the [-ant] distinction. It is possible that altered acoustic reflexes are an obstacle against perfectly identifying the components of sound and assimilating these sounds into the phonological system.

Nerve fiber myelination is caudal to cephalic; thus, brainstem-related auditory abilities are myelinated earlier than those that require the participation of cortical regions; and the acoustic reflex pathways are located in the brainstem.^16^, ^37^

The experiences of children during the initial phases of life - especially in the first year - are decisive for the beginning of speech.^38^ If an anatomical structure that specializes in reading acoustic signals is reacting poorly, there will be a degree of sensory deprivation that may cause the nervous system to reorganize itself because of neuroplasticity, a fundamental cerebral process.^39^ This may be the path of phonologically disordered children during the first phases of speech acquisition.

Mental representations of kinesthetic and auditory sensations need to be exercised for phonemes to be produced. If such production takes place incorrectly, the kinesthetic sensation may cause the erroneously produced phoneme to remain.^40^

This may explain why phonologically disordered children use wrong rules for producing certain phonemes; they are, however, able to perceive and understand these same phonemes in spontaneous conversation. Children gather experience as they grow, so logically their maturity will lead to improved discrimination and understanding of speech patterns.^41^, ^42^

Thus, the participation of acoustic reflexes at the initial stages of child development and its relation with auditory processing make it possible for children to correctly discriminate phonemes and to incorporate them in discourse.

As presented above, phonological system expression, acoustic reflex activation, and auditory processing are intimately linked. If any of these is impaired, the other two will be correspondingly affected, resulting in a lower communication potential and impaired use and assimilation of the phonological system.

## CONCLUSION

Altered auditory processing was found only in phonologically disordered cases.

All phonologically disordered children had altered acoustic reflexes.

The voiced phonemes are more affected than the voiceless phonemes when acoustic reflexes are absent.

The severity of the mean phonological disorder correlated significantly with children that presented augmented acoustic reflexes only. Values were significant in moderately-severe and severe degrees when acoustic reflexes were absent.
